# Aroma-Active Compounds in Robusta Coffee Pulp Puree—Evaluation of Physicochemical and Sensory Properties

**DOI:** 10.3390/molecules26133925

**Published:** 2021-06-27

**Authors:** Nina Buck, Daria Wohlt, Anne Ruth Winter, Eva Ortner

**Affiliations:** 1 Fraunhofer Institute for Process Engineering and Packaging IVV, Giggenhauser Straße 35, 85354 Freising, Germany; daria.wohlt@ivv.fraunhofer.de (D.W.); anneruth.winter@web.de (A.R.W.); eva.ortner@ivv.fraunhofer.de (E.O.); 2 Chair of Aroma and Smell Research, Department of Chemistry and Pharmacy, Friedrich-Alexander-University Erlangen-Nürnberg, Henkestraße 9, 91054 Erlangen, Germany; 3 Institute of Food Technology, Weihenstephan-Triesdorf University of Applied Sciences, Am Staudengarten 11, 85354 Freising, Germany

**Keywords:** aroma extract dilution analysis, by-product, *Coffea canephora*, coffee cherry, gas chromatography-olfactometry, residue, sustainability

## Abstract

Wet coffee processing generates a large amount of coffee pulp waste that is mostly disposed of in the processing units. To reduce this waste and the associated environmental burden, an alternative strategy would be to exploit the coffee pulp to produce a durable and stable consumable product. Accordingly, a puree produced from Robusta coffee pulp was investigated in relation to its physicochemical and sensory properties. After thermal and chemical stabilization, the obtained puree (pH 3.6) was found to exhibit a multimodal particle size distribution, shear-thinning behavior, and lower discoloration, as well as an antioxidant capacity of 87.9 µmol_TE_/g_DM_. The flavor of the puree was examined by sensory evaluation and the corresponding analyses of aroma-active volatile compounds, as determined using aroma extract dilution analyses (AEDA) and gas chromatography-mass spectrometry/olfactometry (GC-MS/O). The puree was characterized by dominant *fruity* (4.4)*, floral* (3.4), *citrusy* (3.3) and *hay-like* (3.3) odor impressions. The aroma-active compounds were predominantly aldehydes, acids, and lactones, whereby (*E*)-*β*-damascenone, geraniol, 4-methylphenol, 3-hydroxy-4,5-dimethylfuran-2(*5H*)-one, and 4-hydroxy-3-methoxybenzaldehyde exhibited the highest flavor dilution (FD) factor (1024), thereby indicating their high impact on the overall aroma of the puree. This study demonstrates an approach to stabilize coffee pulp to produce a *sweet, fruity* puree with comparable physical properties to other fruit purees and that can be used as a new and versatile flavoring ingredient for various food applications.

## 1. Introduction

Coffee is one of the most traded commodities in the world. The annual world coffee consumption was estimated to be over 10,000 t in 2019/2020 [1]. Coffee processing produces large quantities of by-products, mainly coffee pulp, which is typically disposed of as waste [2]. Coffee pulp amounts to 28.7% of the dry weight [3] and 40–50% of the fresh weight of coffee berries [2]. After harvesting, the coffee fruit is subjected to different processing steps for separating the bean from the fruit [2,4]. For dry processing, the fruits are spread out and sundried, hulled with a peeling machine and cleaned, leaving the coffee husks as by-products. For wet processing methods (“pulped natural,” “semi-washed,” and “fully washed”), the pulp and skin are removed mechanically. Then, the mucilage is removed mechanically or by fermentation during the “semi-washed” and “fully washed” processes, respectively, whereas for the “pulped natural” coffee, all mucilage is left on the parchment skin [2,4].

The improper disposal of coffee by-products poses environmental risks by contaminating the water and soil around the processing units [5,6]. As recently reviewed, caffeine and the tannins contained in the coffee fruit are toxic to several aquatic organisms, while the contained chlorogenic acid has negative effects on seed germination and plant growth [6]. Some approaches for the valorization of coffee by-products are its usage as a mushroom cultivation substrate; the production of enzymes, biofuel, and organic acids; and the extraction of bioactive compounds and dietary fiber [6,7]. However, these approaches use only part of the available quantity and are technically not very efficient [8]. The pulp is rich in tannins and caffeine [5], limiting the use of coffee pulp for animal feed due to its anti-nutritive effects [3,6]. However, these phytochemicals have properties that are beneficial to human health. For example, caffeine has been shown to reduce the risk of obesity and type 2 diabetes mellitus, while tannins show anti-inflammatory and antimicrobial effects [5,6]. Recently, the antioxidant and antidiabetic effect of aqueous *Coffea arabica* L. pulp extract was confirmed in in vivo and in vitro models [9]. Moreover, the pulp is rich in nutrients such as carbohydrates, proteins, minerals (especially potassium) and polyphenols [5]. This makes the utilization of coffee pulp as a functional food or food ingredient attractive. Furthermore, so called ‘superfoods’ (healthy, innovative, exotic and/or functional foods) have become popular in recent years and influence consumers’ purchasing decisions [10,11].

Coffee berries already found usage more than a thousand years ago when the pulp of fermented berries was used in Arabia to produce wine, known as qishr [12]. Nowadays, qishr is known as an infusion of spiced coffee husks and it is drunk instead of coffee [12,13]. Elsewhere, the infusion of dried coffee pulp is known as cascara (Spanish for husk) or coffee cherry tea [13,14]. In the European Union (EU), coffee cherry tea is categorized as a “novel food” [4,15]. An application for authorization to place coffee husks from *Coffea arabica* L. on the market in the EU for use as an ingredient in non-alcoholic, water-based beverage infusions is currently under review [16].

Recently, a study investigated the sensory properties of Arabica coffee pulp cold brews compared to coffee seed brews by means of quantitative descriptive analysis (QDA) [17]. The brews obtained from the coffee pulp were rated higher in intensity in their *botanic* (characteristic odor associated with typical dried black tea notes) and *fruity* notes, as well as more *sweet, sour* and *botanic* in taste. In contrast, the brews obtained from coffee seeds elicited higher intensities regarding their *earthy* odor and *earthy*, *bitter* and *sour-roasted* taste impressions [17]. Moreover, the aroma-active compounds in different cascara samples were recently evaluated using AEDA and identified by means of gas chromatography-olfactometry (GC-O) and gas chromatography-mass spectrometry (GC-MS) [18].

Coffee pulp has also been used to develop a new food ingredient: coffee flour. It consists of a high fiber and ash content (18% and 8%, respectively), is low in fats (1.6%) [19] and has been proposed for use in breads, cookies, muffins, squares, brownies, pasta, sauces, and beverages. The taste of the coffee flour was described as floral, citrus and roasted-fruit, rather than exhibiting a coffee taste [12]. Other proposed applications for coffee pulp in food products or ingredients are in jams, juices, concentrates, jelly, and alcoholic beverages [20,21]. In the case of coffee pulp juice, the addition of 15–20% sugar improved panelists preferences compared to the 10% sugar concentration [22]. Despite this interest, the sensory properties and key aroma compounds of coffee pulp itself have not been hitherto investigated via a systematic approach [17], even though aroma-active compounds are important flavor contributors and therefore parameters for consumer acceptance.

In the present approach, Robusta coffee pulp was microbiologically and enzymatically stabilized to produce a fruit puree for its potential application as a new and versatile food ingredient. The aim of this work was to characterize the physicochemical properties of the coffee pulp puree, to identify its key aroma-active volatile compounds, and to compare the analytical data with the sensory profiles determined by QDA. The aroma-active volatiles in the fruits were characterized by GC-O with AEDA [23,24,25,26,27], and the chemical identity of individual compounds was determined by GC-O/MS.

## 2. Results and Discussion

### 2.1. Characterization of Robusta Coffee Pulp Puree

#### 2.1.1. Characterization of Native Coffee Pulp Puree

The native Robusta (*C. canephora*) pulp puree, which was pasteurized during comminution, had a pH of 5.0, a dry matter content of 6.3%, and a total soluble solids content of 0.5 °Brix (Table 1). The native puree showed L*, a* and b* values of 32.6, 15.0 and 17.5, respectively. The antioxidant capacity, measured by DPPH assay, was 77.3 µmol_TE_/g_DM_, equaling to 19.3 mg_TE_/g_DM_. The observed antioxidant capacity was higher than 0.6–2.24 mg_TE_/g_DM_ obtained for dried Robusta coffee pulp [28], which might be explained by the variability of the raw materials (e.g., cultivar, maturity, cultivation conditions) or differences among the stabilizing processes. 

**Figure 1:**
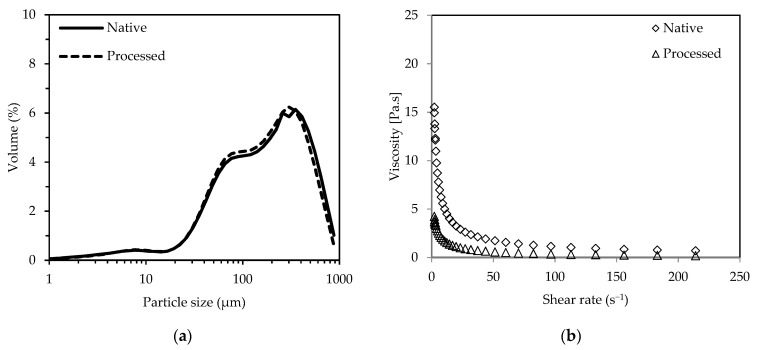
Particle size distribution (**a**) and apparent viscosity over a shear rate range from 2.14 to 214 s^−1^ (**b**) of native and processed Robusta coffee pulp purees.

As indicated by laser scattering analysis, the native puree particles showed a multimodal size distribution (Figure 1a). The respective peaks were around 200 µm, 80 µm, and 8 µm. While the large and intermediate particles possibly consisted of cell aggregates and single parenchyma cells, as previously found in jaboticaba and apple puree [29,30], cell wall fragments could account for the small particles. The native coffee pulp puree showed shear-thinning behavior (Figure 1b), as commonly exhibited by other fruit purees [29,30,31].

Since the application of 2% citric acid (*w/w*) and thermal treatment successfully preserved pear puree color by inhibiting peroxidase and polyphenol oxidase [32], a similar process was applied to the Robusta coffee pulp. However, due to the results from preliminary tests and its acidic taste, the citric acid concentration was limited to 1% (*w*/*w*).

#### 2.1.2. Comparison of Native and Processed Robust Coffee Pulp Puree

Besides a native Robusta coffee pulp puree (pasteurized), a processed puree was produced through blanching of the coffee cherries and the addition of citric acid. Representative images of the purees can be found in the report by Martins Moreira et al. [33]. The processed puree was also characterized for its physicochemical properties (Table 1). By adding 1% (*w*/*w*) citric acid, the pH of the processed puree was reduced to 3.6. The dry matter content of the processed puree was 5.5%, with a total soluble solids content of 0.88 °Brix, indicating no significant changes (*p* ≥ 0.095).

Comparing the colors of the native and the processed purees, improved color retention in the processed puree was achieved by preliminary blanching of the cherries and the addition of citric acid. This was reflected in the significantly higher L* (brightness), a* (red), and b* values (yellow) of the processed puree (*p* ≤ 0.006). Additionally, a significant increase in the antioxidant capacity to 87.9 µmol_TE_/g_DM_ was observed in the processed puree (*p* = 0.018). Since coffee cherries are known to be prone to color degradation caused by the enzymatic and non-enzymatic oxidation of phenolic compounds like anthocyanins [34], less discoloration of the processed puree was expected. Based on the higher antioxidant capacity after processing, this color retention might result from the better retention of anthocyanins. Similar observations have been reported previously, as blanching pretreatment positively impacted the retention of anthocyanins and total phenolic content in blueberry puree [35]. The observed antioxidant capacity of the processed puree was higher than in the dried Robusta coffee pulp [28], indicating that the process might represent a more gentle alternative to preserving the coffee pulp. A caffeine content of 2.1 mg/g_DM_ was obtained for a puree produced in a comparable scale-up process [33], which was similar to the caffeine content of dried coffee pulp [28].

The volume mean diameter d(*v*, 0.5) of the processed puree was 164.9 µm. No significant decrease of the particle size could be observed in comparison to the native puree (*p* = 0.774). The particle size characteristics were similar to those of native puree, indicating no further cellular breakdown was caused by the additional processing. The processed puree also showed shear-thinning behavior, with a similar apparent viscosity compared to tart cherry puree over a varying shear rate (0–200 s^−1^) (Figure 1b) [31].

Besides improved color and antioxidant capacity retention, the proposed process might also enhance the microbiological safety of the Robusta coffee pulp puree, since whole coffee cherries and coffee pulp can show high loads of microorganisms like yeast, molds, and mesophilic bacteria [33].

Based on preliminary experiments [33] and in accordance with the literature [22], 19% (*w*/*w*) of sucrose was added to the processed puree for sensory evaluation to balance the acidity and enhance its taste.

### 2.2. Sensory Evaluation

**Figure 2:**
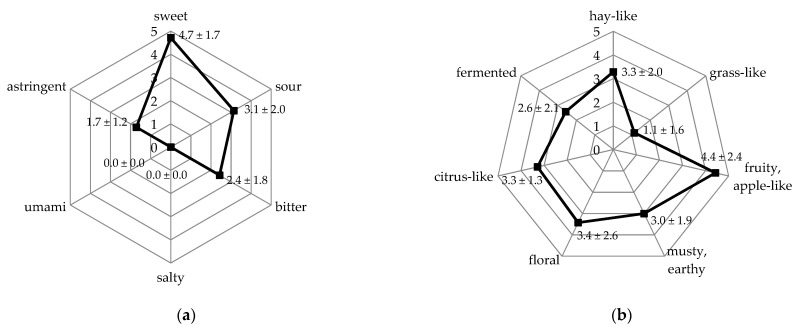
Taste profile (**a**) and aroma profile (**b**) of a processed Robusta coffee pulp puree, perceived via retronasal evaluation. Note that the intensity scale has been adjusted to cover the range of rated intensities (maximum rated intensity of 4.7), rather than the full range available (10).

The retronasal aroma and taste properties of the Robusta coffee pulp puree were evaluated by rating the different impressions on a linear scale from 1 (weak) to 10 (strong). The resulting retronasal aroma profile and the taste profile are displayed in Figure 2. The highest intensity of a single impression was rated with 4.7 for the *sweet* taste perception. The dominance of the *sweet* taste perception can be explained by the high sucrose concentration (19%). Moreover, the taste was described as *sour* (3.1), *bitter* (2.4), and *astringent* (1.7). To describe the retronasal aroma properties, the *fruity, apple-like* impression was rated with the highest intensity (4.4), followed by *floral* (3.4), *hay-like* (3.3), *citrus-like* (3.3), *musty, earthy* (3.0), *fermented* (2.6), and *grass-like* (1.1) notes. The predominant odor impressions of a cold brew Arabica pulp tea were *botanic* and *fruity*, associated with typical dried black tea notes and floral, sweet, and ripe fruit notes characteristic of coffee pulp, respectively [17]. Moreover, an *earthy* impression, associated with bread-like and wet soil-like notes, was perceived. This shows that for both coffee pulp puree and tea, the *fruity* odor is predominant, together with *hay-like* or *botanical* notes. Moreover, *earthy* impressions contribute to the overall aroma profile of both products.

### 2.3. Identification of Aroma-Active Compounds in Unprocessed and Processed Coffee Pulp Puree

The aroma-active volatiles responsible for the overall aroma impression in the coffee pulp puree and the unprocessed pulp were isolated by dichloromethane (DCM) extraction, followed by solvent-assisted flavor evaporation (SAFE)-distillation. By comparing the overall odor of the final distillates with the overall odor of the original samples, successful extraction of the target odorants could be confirmed. Then, the distillates were subjected to AEDA by means of GC-O analyses and a total of 55 aroma-active regions were detected within an FD factor range of 2 to 2048, eliciting *fruity, flowery, seasoning-like, fatty, green* and *fecal* odor qualities, amongst others (Table 2). 

The highest flavor dilution (FD) factor determined in the processed coffee pulp puree was 1024, which was exhibited by the aroma compounds (*E*)-*β*-damascenone (*apple juice-like, grape juice-like*), geraniol (*flowery*), 4-methylphenol (*horse stable-like, fecal*), 3-hydroxy-4,5-dimethylfuran-2(*5H*)-one (*lovage-like, celery-like*), and 4-hydroxy-3-methoxybenzaldehyde (*vanilla-like*), followed by an unknown, *flowery, hay-like* smelling compound (at RI 1720 on DB-FFAP and RI 1030 on DB-5), the *lovage-like, seasoning-like* smelling 3-hydroxy-2-pyrone, an unknown, *broth-like* smelling compound (at RI 2340 on DB-FFAP) and the *beeswax-like, honey-like* smelling phenylacetic acid exhibited FD factors of 512. The *flowery* smelling linalool, the *fatty* smelling (*E,E*)-2,4-decadienal and the *musty, coriander-like, fatty* smelling octanoic acid were determined with FD factors of 256. The 45 identified aroma compounds belonged to diverse chemical groups that were predominantly aldehydes, but included acids, lactones, phenols, ketones, alcohols, terpenes, pyrazines, and esters. The precursors of carbonyl compounds, such as aldehydes, ketones, alcohols, and esters, are amino acids and fatty acids. The oxidative cleavage of linoleic and linolenic acid especially lead to the formation of these carbonyl compounds [36]. Lactones can be formed by the oxidation and subsequent cyclization of oleic and linoleic acids, while phenols can be formed by thermal or microbial degradation of phenolic acids or lignin [36]. Terpenes and terpenoids are mainly formed via the mevalonate pathway [37]. In fruit juices, terpenes are predominantly present as glycosides [36]. In comparison, the aroma of apples and pears is dominated by aliphatic esters, alcohols, and aldehydes, while the aroma-active compounds in strawberries, raspberries, and blackberries may be similar to those in apples or pears, with additional ketones and lactones [38]. However, the aroma of citrus fruits is dominated by terpenes, while the fruity perception of tropical fruits derives from sulfur compounds at low concentrations in a matrix of high sugars and low acids [38]. 

AEDA was recently performed for a hot Arabica pulp infusion, whereby (*E*)-*β*-damascenone and 3-hydroxy-4,5-dimethylfuran-2(5*H*)-one were determined as having the highest FD factor, followed by 2-methoxyphenol [18]. The majority of the aroma-active compounds identified in the Robusta pulp puree and the Arabica pulp infusion were similar. Some of these substances were also reported in the headspace of fresh Robusta and Arabica cherries [39]. However, the samples were not investigated using an olfactometric approach and therefore, no conclusion on the aroma activity of the dominating terpenes and sesquiterpenes identified of fresh coffee cherries can be drawn [39,40].

The identified compounds are in agreement with the descriptive aroma profile analysis: the *fruity, apple-like* impression was ranked highest (4.4), which might reflect the presence of the *apple juice-like, grape juice-like* smelling (*E*)-*β*-damascenone (FD 1024), as well as an unknown, *fruity, apple juice-like* smelling substance. Moreover, *δ*-dodecalactone (FD 32), *γ*-decalactone (FD 8) were identified as *fruity* smelling substances, which could have contributed to this aroma characteristic. The *floral* impression (rated with 3.4) might be explained by the *flowery* smelling geraniol (FD 1024), linalool (FD 256), *β*-ionone (FD 128), and phenylacetaldehyde (FD 64). The *citrus-like* impression (rated with 3.3) might be explained by the *citrus-like* smelling substances 2-heptanol (FD 128), (*E,E*)-2,4-octadienal (FD 64), octanal, and nonanal (both FD 16). The *musty, earthy* odor impression was rated with an intensity of 3.0. As a *musty* smelling substance, octanoic acid was identified with an FD factor of 256. The odor of pyrazines, such as 2-isobutyl-3-methoxypyrazine (FD 128) and 2-*sec*-butyl-3-methoxypyrazine (FD 2), might also be described as *earthy*. The *grass-like* odor impression (rated with 1.1) might have been elicited by *green* smelling aldehydes, such as (*E*)-2-nonenal (FD 64), (*E,E*)-2,4-octadienal (FD 64), (*E,Z*)-2,4-decadienal (FD 16) and (*E,E*)-2,4-heptadienal (FD 2), and *grassy* smelling substances, such as (*Z*)-3-hexenol (FD 16), (*E*)-2-octenal (FD 4), hexanal (FD 2), and an unknown substance (FD 2). Moreover, a *fermented* impression was perceived during sensory evaluation with an intensity of 2.6. However, there was no odor-active region perceived during GC-O that smelled explicitly *fermented*. 

It is noteworthy that odor impressions are complex mixtures of many aroma-active and odorless volatile substances, present at different concentrations [41]. Synergistic, additively or suppressive effects that follow unpredictable rules can occur, thus linear correlations between a single substance and an olfactory stimulus cannot be drawn [41,42]. For further investigation of the overall aroma and key aroma compounds, quantification followed by omission experiments and recombination models need to be conducted [41]. Overall, 55 aroma-active areas were detected by a systematic approach, 45 thereof were unequivocally identified and reported in coffee pulp for the first time.

**Figure 3:**
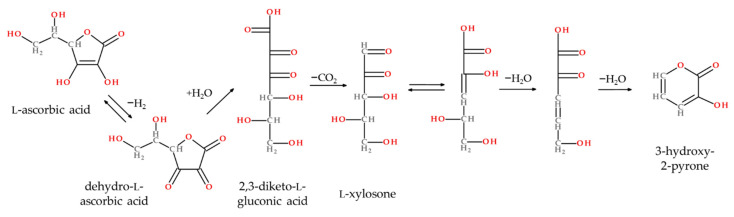
Formation of 3-hydroxy-2-pyrone from l-ascorbic acid (modified from [47,48]).

Moreover, the distillate obtained from unprocessed coffee pulp was analyzed by means of GC-O, followed by identification experiments to investigate whether the aroma-active compounds from the pure coffee pulp were affected by the processing of the puree. The majority of the aroma-active volatiles were similar in both identity and FD factors. Interestingly, 3-hydroxy-2-pyrone (FD 512) was not perceived (FD < 1) in the pure coffee pulp distillate. 3-Hydroxy-2-pyrone has been shown to be a degradation product of ascorbic acid in heated aqueous solutions [43] and in thermally treated fruit samples such as strawberry jam, acerola fruit, and cloudberry juice [44,45,46]. Under aerobic and acidic conditions, ascorbic acid is oxidized to dehydroascorbic acid, which is unstable in aqueous solutions and forms 2,3-diketo-l-gluconic acid by cleavage of the lactone ring and the addition of water (Figure 3). 3-Hydroxy-2-pyrone is then formed by further decarboxylation, intermolecular redox reactions, and dehydrations [47,48]. Most likely, in the present sample the compound was formed by thermal treatment and the addition of citric acid during the preparation of the coffee pulp puree.

Geraniol was perceived with a lower FD factor in the pure coffee pulp distillate (FD 16), than in the puree (FD 1024). This might be explained by a release of geraniol through acidic hydrolysis during the thermal processing of the puree. In fruits, terpenes such as geraniol are often found to be glycosidic: bound and released by enzymatic or acidic hydrolysis [36]. Geraniol was also identified in a mixture of the pulp and wastewater from the depulping and demucilage processing of the coffee beans (*Coffea arabica* L., variety Catuaí 99 vermelho) [49]. An increase in its concentration was observed after fermentation with yeast (*Haseniaspora uvarum* UFLA CAF76) [49].

## 3. Materials and Methods

**Figure 4:**
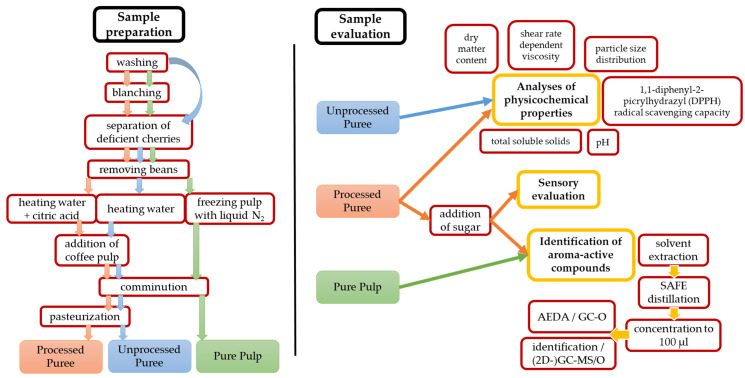
Schematic overview of the methodology used for this study.

A schematic overview of the methodology applied for this study is given in Figure 4.

Information on the reference aroma compounds and additional chemicals used in the analyses is provided in the Supplementary Material.

### 3.1. Preparation of Coffee Pulp Puree

The coffee pulp puree was made from ripe, frozen Robusta coffee (*Coffea canephora*) cherries from Brazil (harvested in 2019, experimental field at the Agronomic Institute of Campinas (IAC) in Campinas, São Paulo, Brazil). After defrosting, the cherries were washed and blanched for 1.5 min in boiling water. Dry or green cherries were separated manually and not used for puree production. The coffee beans were subsequently removed by hand. Tap water (1.5 times the mass of the coffee pulp) and citric acid (1% *w*/*w*, referred to the sum of the mass of the coffee pulp and the water) were heated for 5 min at 100 °C in a knife mill (TM31, Vorwerk, Wuppertal, Germany) while mixing at level 2. The coffee pulp was added, ground (level 10, 80 °C, 5 min) and pasteurized (level 0.5, 80 °C, 5 min). The resulting puree represented the processed puree. For comparison, a native coffee pulp puree was produced without the blanching step. No citric acid was added during comminution. For the sensory and aroma analyses, sucrose (19% *w*/*w*, also referred to as the sum of the mass of the coffee pulp and the water) was added after the processed puree was chilled to room temperature.

### 3.2. Physicochemical Analyses

The following parameters characterized the coffee pulp puree in terms of its physicochemical properties: dry matter content, total soluble solids, pH, color, 1,1-diphenyl-2-picrylhydrazyl (DPPH) radical scavenging capacity, particle size, and shear rate dependent viscosity. Dry matter content was determined gravimetrically by drying the sample at 105 °C with a moisture analyzer MA100 (Sartorius Lab Instruments GmbH & Co. KG, Göttingen, Germany) until constant weight was achieved. Total soluble solids were determined using a digital handheld refractometer DR301-95 (A. Krüss Optronic GmbH, Hamburg, Germany). The DigiEye color imaging system (DigiEye V2.62, VeriVide, Leicester, UK) was used for color measurements, comprising an illumination box with diffuse illuminant D65 and a Nikon D90 digital camera. Digitizer calibration charts were used to calibrate the system. For the color measurement, the puree was evenly distributed in a Petri dish and the average surface color was expressed as CIE L*a*b*-values with L*, a* and b* ranging from black [−] to white [+], from green [−] to red [+], and from blue [−] to yellow [+], respectively. To determine DPPH radical scavenging activity, an acetone extract was prepared. Therefore, 20 g (±0.01 g) of the puree was extracted in 60 mL of chilled acetone at 4 °C for 10 min by an UltraTurrax T25 (IKA Werke GmbH & Co. KG, Staufen, Germany). After vacuum filtration, the sample was diluted up to a final volume of 100 mL in a volumetric flask, and stored at −18 °C under nitrogen until analysis. The antioxidant capacity was measured according to a DPPH radical scavenging assay, as described elsewhere [50], with some modifications. The extract was mixed with 96 µM DPPH in ethanol (instead of methanol). After incubating for 20 min, the absorbance was measured in a spectrophotometer (µQuant MQX 200, BioTek Instruments, Winooski, VT, USA). Ethanolic Trolox solutions in the concentration range of 10–1000 µmol/L were used to obtain a calibration curve. The antioxidant activity was expressed as µmol Trolox equivalents (TE) on a dry matter basis. The particle size distribution of the purees was determined by a Mastersizer S long bed Version 2.15 laser diffraction particle size analyzer (Malvern Instruments Ltd., Malvern, UK) equipped with a 300 RF lens. A few droplets of the sample were dispersed in demineralized water in a dispersion unit at 3000 rpm. Four readings were performed for each sample, and the particle size distribution and the volume mean diameter d(*v*, 0.5) were determined using Mie theory. Flow curves were measured at 20 °C using a Physica MCR 301 rotational rheometer (Anton Paar Germany GmbH, Ostfildern-Scharnhausen, Germany) equipped with PP50 parallel-plate geometry. The shear rate was increased from 2.14 to 214 s^−1^ within 10 min, and sample viscosity was measured.

### 3.3. Sensory Evaluation

#### 3.3.1. Panelists

The sensory evaluation was performed by seven trained panelists from the Department of Sensory Analytics of the Fraunhofer Institute for Process Engineering and Packaging IVV (Freising, Germany). Panelists exhibited normal olfactory function and had no known illnesses at the time of examination. Trainings were carried out weekly based on an in-house established odor-language with selected references, corresponding to a total of about 150 different odorants.

#### 3.3.2. Aroma Profile Analysis

Sensory evaluation was performed according to DIN EN ISO 13299:2016 (general guidance for establishing a sensory profile). For this evaluation, 20 g (±1 g) of the coffee pulp puree was presented in 140 mL covered glass vessels in a sensory room at 21 °C. Aroma qualities were determined in consensus by the panel. The following corresponding odorants or products were provided as references: *hay-like* (hay), *grass-like* (hexanal), *fruity, apple-like* ((*E*)-*β*-damascenone), *musty, earthy* (2,3-diethyl-5-methylpyrazine), *flowery* (linalool), *citrus-like* (octanal). The sample was evaluated in direct comparison by rating the intensity of the selected attributes and their overall intensity on a scale from 0 (no perception) to 10 (strong perception). The results were averaged and the mean values were plotted in a spider-web diagram.

### 3.4. Isolation of Volatiles

The pure pulp was frozen with liquid nitrogen and subsequently ground with a commercial blender (1 L, Waring Commercial, Torrington, CT, USA). Coffee pulp puree and pure coffee pulp (150 g ± 1 g and 50 g ± 1 g, respectively) were extracted with DCM (350 mL) by stirring vigorously for 2 h at room temperature in a closed vessel. Afterwards, the extracts were filtered, and the volatiles were separated from the non-volatiles using the solvent-assisted flavor evaporation technique [51]. The obtained distillates were dried over anhydrous sodium sulfate, filtered, and concentrated at 50 °C to ∼3 mL by a Vigreux column (50 cm × 1 cm i.d.), and finally reduced to ∼100 µL by microdistillation [52].

### 3.5. Aroma Extract Dilution Analyses

The FD factors of the single odorants were determined using GC-O, representing the last dilution in which the individual odorant was still perceivable [53]. The aroma distillate (cf. Section 3.4) was diluted stepwise (1+1, *v*+*v*) with DCM, resulting in solutions corresponding to FD factors up to 2048. GC-O was performed on the undiluted distillate by three trained panelists (cf. Section 3.3.1) to avoid potentially overlooking odorants, for instance due to partial anosmia or lower sensitivities to particular substances. A blank sample obtained by applying the identical work-up procedure underwent similar analyses.

### 3.6. Gas Chromatography-Olfactometry

GC-O was performed with a Trace GC Ultra (Thermo Fisher Scientific GmbH, Dreieich, Germany) using DB-FFAP and DB-5 (both 30 m × 0.32 mm, film thickness 0.25 µm; J&W Scientific, Agilent Technologies GmbH, Waldbronn, Germany) capillary columns. Furthermore, the system consisted of a precolumn (uncoated, deactivated fused silica capillary, 3–5 m × 0.32 mm) and a Y-splitter after the main column, splitting the effluent in a 1:1 volume ratio and transferring it to the flame ionization detector (FID) and the olfactory detection port (ODP) via two uncoated, deactivated fused silica capillaries (0.7 m × 0.2 mm). Aliquots (2 µL) of the undiluted distillate and the dilutions were injected manually using the cold on-column technique (40 °C). After 2 min, the temperature was raised at 8 °C/min to 235 °C (DB-FFAP) or 250 °C (DB-5), respectively, and held for 5 min. The flow rate of the carrier gas helium was 2.2 mL/min. The temperature of the FID and the ODP were set to 250 °C and 230 °C, respectively. Linear retention indices (RIs) of the aroma compounds were calculated [54].

### 3.7. Gas Chromatography-Mass Spectrometry/Olfactometry

GC-MS/O was performed with a Trace GC Ultra and a Trace DSQ mass spectrometer (both Thermo Fisher Scientific GmbH) using DB-FFAP and DB-5 (both 30 m × 0.32 mm, film thickness 0.25 µm; J&W Scientific) capillary columns. The aroma distillate was injected by an MPS 2 multipurpose sampler (Gerstel GmbH & Co. KG, Mülheim an der Ruhr, Germany) using the cold on-column technique (40 °C). The same temperature program as described in Section 3.6. was applied. The flow rate of the helium carrier gas was 2.3 mL/min. At the end of the capillary column, the effluent was split and led to an ODP (250 °C) and the DSQ mass spectrometer, using deactivated fused silica capillaries (0.5 m × 0.2 mm). Mass spectra were generated in electron ionization (EI) full scan mode (*m*/*z* range 35–350, 70 eV).

### 3.8. Two-Dimensional Gas Chromatography-Mass Spectrometry/Olfactometry

The 2D-GC-MS/O system consisted of two Varian CP 3800 gas chromatographs (Agilent Technologies GmbH), connected with a CTS 1 cryo trap system (Gerstel GmbH & Co. KG). Aliquots of the sample distillates (2 µL) were automatically applied using the cold on-column-technique at 40 °C using an MPS 2XL multipurpose sampler (Gerstel GmbH & Co. KG). After 2 min, the temperature was raised at 8 °C/min to 235 °C and held for 5 min in the first GC oven. Odorants of interest were cryo trapped (−100 °C) using a MCS2 multi-column switching system (Gerstel GmbH & Co.KG). After thermodesorption (250 °C), the cryo trapped volatiles were transferred into the second GC system, using a starting temperature of 40 °C. Then, the temperature was raised at 8 °C/min to 250 °C and held for 1 min. The helium carrier gas flow was 8 mL/min.

The first GC system was equipped with an uncoated fused silica capillary as a precolumn (2–3.5 m × 0.53 mm) and a DB-FFAP column (30 m × 0.32 mm, film thickness 0.25 µm; J & W Scientific), the second system with a DB-5 column (30 m × 0.32 mm, film thickness 0.25 µm; J & W Scientific). At the end of the capillary column of the first system, the effluent was split into equal parts and led to an ODP and an FID (each 250 °C). At the end of the second GC system, the effluent was split again into two equal parts to a second ODP and a Saturn 2200 Ion Trap mass spectrometer (Agilent Technologies GmbH), operating in EI full scan mode (*m*/*z* range 35–399, 70 eV).

### 3.9. Statistical Analysis

The results of the physicochemical characterization of the Robusta coffee pulp puree were expressed as mean ± standard deviation (SD). Statistical analysis was performed using Welch’s unequal variances *t*-test (*p* < 0.05) to determine significant differences among two groups using Sigma Plot 14.0 (Systat Software Inc., San Jose, CA, USA). 

## 4. Conclusions

The utilization of coffee residue as food products is still limited, to date. By applying a blanching step and adding citric acid, an enzymatically and color-stable coffee pulp puree was produced with an antioxidant capacity of 87.9 µmol_TE_/g_DM_. Evaluation of the sensory profile of the puree revealed that it was dominated by *fruity, floral, citrusy* and *hay-like* odor impressions, as well as *sweet, sour* and *bitter* tastes. Overall, 55 aroma-active areas were detected during AEDA, 45 of which were unequivocally identified by GC-MS/O and are presently reported for the first time in coffee pulp. The highest FD factor of 1024 was obtained for the compounds (*E*)-*β*-damascenone (*apple juice-like, grape juice-like*), geraniol (*flowery*), 4-methylphenol (*horse stable-like, fecal*), 3-hydroxy-4,5-dimethylfuran-2(*5H*)-one (*lovage-like, celery-like*), 4-hydroxy-3-methoxybenzaldehyde (*vanilla-like*), 3-hydroxy-2-pyrone (*lovage-like, seasoning-like*), and phenylacetic acid (*beeswax-like, honey-like*).

This systematic study demonstrates that coffee pulp puree is a diverse functional food or functional food ingredient that has high potential on the food market because of its pleasant aroma impressions, high antioxidant capacity, and caffeine content. As we were able to produce a puree with similar physicochemical properties to those described for other fruit purees, applications in smoothies, yogurts, fillings, and similar products are conceivable. By incorporating the whole coffee pulp into food products in the form of a puree rather than an aqueous extract or individual extracted components, a higher fiber content can be achieved. The results of this study indicate that the production of a coffee pulp puree represents an innovative and holistic approach for the use of Robusta coffee residues.

## Figures and Tables

**Table 1:** Physicochemical properties of native and processed Robusta coffee pulp puree.

Physiochemical Properties	Native	Processed
pH (−)	4.95 ± 0.01 ^A^	3.57 ± 0.05 ^B^
Dry matter content (%)	6.27 ± 0.44 ^A^	5.50 ± 0.60 ^A^
Total soluble solids (°Brix)	0.48 ± 0.18 ^A^	0.88 ± 0.29 ^A^
L* (−)	32.63 ± 0.16 ^B^	36.03 ± 0.38 ^A^
a* (−)	15.03 ± 0.12 ^B^	16.92 ± 0.20 ^A^
b* (−)	17.53 ± 0.22 ^A^	21.64 ± 0.63 ^B^
Antioxidant capacity (µmol_TE_/g_DM_)	77.26 ± 0.36 ^B^	87.93 ± 3.95 ^A^
d(*v*, 0.5) (µm)	167.76 ± 1.67 ^A^	164.94 ± 2.50 ^A^

Values are expressed as mean ± SD of two experiments. Means in the same row with no letter in common differ significantly (*p* < 0.05).

**Table 2:** Aroma-active compounds and corresponding flavor dilution (FD) factors identified in the distillate obtained from the processed Robusta coffee pulp puree and from the unprocessed Robusta coffee pulp.

RI ^a^		Compound ^b^	Odor Quality ^c^	FD ^d^ Factor	
DB-FFAP	DB-5			Puree	Unprocessed Pulp
1809	1387	(*E*)-*β*-damascenone	*apple juice-like, grape juice-like*	1024	1024
1833	1255	geraniol	*flowery*	1024	16
2078	1086	4-methylphenol	*horse stable-like, fecal*	1024	256
2185	1102	3-hydroxy-4,5-dimethylfuran-2(*5H*)-one ^f^	*lovage-like, celery-like*	1024	512
2563	1400	4-hydroxy-3-methoxybenzaldehyde	*vanilla-like*	1024	≥2048
1720	1030	unknown	*flowery, hay-like*	512	256
1966	992	3-hydroxy-2-pyrone	*lovage-like, seasoning-like*	512	<1
2340	n.d. ^e^	unknown	*broth-like*	512	512
2541	1254	phenylacetic acid	*beeswax-like, honey-like*	512	256
1534	1104	linalool	*flowery*	256	256
1802	1317	(*E,E*)-2,4-decadienal	*fatty*	256	512
2041	1175	octanoic acid	*musty, coriander-like, fatty*	256	256
1301	955	2-heptanol	*coconut-like, citrus-like*	128	16
1507	1178	2-isobutyl-3-methoxypyrazine	*bell pepper-like, pea-like*	128	256
1650	859	2/3-methylbutanoic acid	*cheesy, sweaty, banana-like*	128	256
1759	1196	methyl salicylate	*eucalyptus-like, solvent-like*	128	512
1930	1488	*β*-ionone	*violet-like, flowery*	128	256
1853	1087	2-methoxyphenol	*smoky, smoked ham-like*	64	8
1292	979	1-octen-3-one	*mushroom-like*	64	128
1445	905	3-(methylthio)propanal	*cooked potato-like*	64	16
1523	1160	(*E*)-2-nonenal	*fatty, cardboard-like, green*	64	64
1579	1106	(*E,E*)-2,4-octadienal	*fatty, green, citrus-like*	64	1024
1638	1050	phenylacetaldehyde	*beeswax-like, rapeseed-like, flowery*	64	256
1690	1212	(*E,E*)-2,4-nonadienal	*fatty, nutty*	64	4
2019	1360	*γ*-nonalactone	*coconut-like*	64	128
2156	1363	eugenol	*clove-like*	64	256
2250	n.d. ^e^	unknown	*phenolic, gas-like*	64	64
2447	1303	indole	*faecal*	64	<1
2411	1705	*δ*-dodecalactone	*peach-like*	32	16
1231	896	(*Z*)-4-heptenal ^f^	*fishy, fatty*	32	<1
1413	n.d. ^e^	unknown	*licorice-like, aniseed-like, fatty*	32	<1
1280	1002	octanal	*citrus-like, soapy*	16	16
1327	928	2-acetyl-1-pyrroline ^f^	*popcorn-like, roasty*	16	8
1373	861	(*Z*)-3-hexenol	*green, grassy, metallic*	16	16
1383	1103	nonanal	*soapy, citrus-like*	16	8
1753	1290	(*E,Z*)-2,4-decadienal	*fatty, green*	16	512
1911	1142	*γ*-octalactone	*coconut-like*	16	16
1986	1376	*trans*-4,5-epoxy-(*E*)-2-decenal	*metallic*	16	512
2100	n.d. ^e^	unknown	*faecal, ink-like*	16	<1
1618	808	butanoic acid	*sweaty, cheesy*	8	16
2129	1471	*γ*-decalactone	*peach-like, fruity*	8	128
984	720	2,3-butanedione ^f^	*butter-like*	4	16
1418	1057	(*E*)-2-octenal	*fatty, soapy, grassy*	4	64
1445	619	acetic acid	*vinegar-like*	4	8
1733	n.d. ^e^	unknown	*fruity, apple juice-like*	4	≥2048
2172	1179	3-ethylphenol	*leather-like, ink-like, phenolic*	4	8
1015	n.d. ^e^	unknown	*pungent, musty*	2	2
1062	n.d. ^e^	unknown	*citrus-like, fruity*	2	64
1080	801	hexanal	*grassy*	2	<1
1140	n.d. ^e^	unknown	*green, grassy*	2	8
1494	1016	(*E,E*)-2,4-heptadienal	*fatty, green*	2	<1
1489	1178	2-*sec*-butyl-3-methoxypyrazine	*pea-like*	2	16
1552	854	2-methylpropanoic acid	*cheesy, sweaty*	2	8
1904	1115	phenylethanol	*rosy, flowery*	2	<1
1990	n.d. ^e^	unknown	*fruity, caramel-like*	2	<1

^a^ RI: (linear) retention index. ^b^ Odorants were identified by comparison of their odor quality and intensity, RI on DB-FFAP and DB-5 capillaries, as well as mass spectra with data of the respective reference compounds. ^c^ Odor quality perceived at the odor detection port (ODP) during gas chromatography-olfactometry (GC-O). ^d^ Flavor dilution (FD) factor on a DB-FFAP capillary column. ^e^ n.d.: not determined. ^f^ No unequivocal mass spectrum was obtained; identification is based on the remaining criteria given in footnote b.

## Data Availability

The data presented in this study are available on request from the corresponding author.

## Supplementary Materials

The following are available online, Chemicals and Reference Compounds.

## Sample Availability

Samples of the compounds are not available from the authors.
