# Supplementary material for: Aroma-Active Compounds in Robusta Coffee Pulp Puree—Evaluation of Physicochemical and Sensory Properties
Source: Molecules. 2021 Jun 27;26(13):3925. doi: 10.3390/molecules26133925 (PMC8271582; doi:10.3390/molecules26133925)
Supplement: Supplementary file 1 [file molecules-26-03925-s001.zip › molecules-1266264-supplementary.pdf]

Supplementary Material

Aroma-Active Components in Robusta Coffee Pulp Puree – Evaluation of Physicochemical and Sensory Properties

Nina Buck [1,2,*], Daria Wohlt [1], Anne Ruth Winter [1,3], and Eva Ortner [1]

Materials and Methods

Chemicals and Reference Compounds

A homologues series of the alkanes C6–C26 (Fluka and Sigma-Aldrich, Steinheim, Germany), diluted in pentane (Th. Geyer, Renningen, Germany) was used for determining (linear) retention indices (RIs). Anhydrous sodium sulfate, freshly distilled dichloromethane (Th. Geyer), liquid nitrogen (Linde Gas, Pullach im Isartal, Germany) and sodium chloride (Fluka) were used in sample preparation/extraction procedures, as described in the main text. The following pure reference compounds were used: (*E,Z*)-2,4-decadienal was synthesized according to [1]; 3-hydroxy-2-pyrone (abcr, Karlsruhe, Germany); 3-sec-butyl-2-methoxypyrazine ≥99% (Acros Organic, Geel, Belgium); 2-acetyl-1-pyrroline ≥95%; *trans*-4,5-epoxy-(*E*)-2-decenal ≥97% (aromaLab, Planegg, Germany); 4-allyl-2-methoxyphenol (eugenol) ≥99%; 2,3-butanedione ≥99%; butanoic acid ≥99.5%; (*Z*)-3-hexenol ≥98%; indole ≥98.5%; nonanal ≥95% (Fluka, Steinheim, Germany); 3-ethylphenol ≥98% (Riedel-de-Haen, Seelze, Germany); *δ*-dodecalactone ≥98%; (*E,E*)-2,4-heptadienal ≥88%; 2-methylpropanoic acid ≥99% (SAFC, Steinheim, Germany); 3-(methylthio)propanal (methional) ≥97%; acetic acid ≥99%; (*E*)-*β*-damascenone 1.1 wt-%; (*E,E*)-2,4-decadienal ≥85%; *γ*-decalactone ≥98%; 2,3-diethyl-5-methylpyrazine ≥99%; 3,7-dimethylocta-1,6-dien-3-ol (linalool) ≥97%; (2*E*)-3,7-dimethylocta-2,6-dien-1-ol (geraniol) ≥96%; 2-heptanol ≥97%; (*Z*)-4-heptenal ≥98%; hexanal ≥98%; 4-hydroxy-3-methoxybenzaldehyde (vanillin) ≥99%; 3-hydroxy-4,5-dimethyl-2(5*H*)furanone (sotolone) ≥97%; *β*-ionone ≥97%; 2-isobutyl-3-methoxypyrazine ≥99%; 2-methoxyphenol ≥99%; methyl salicylate ≥99%; 3-methylbutanoic acid ≥99%; 4-methylphenol (*p*-cresol) ≥98%; (*E,E*)-2,4-nonadienal ≥85%; *γ*-nonalactone ≥98%; (*E*)-2-nonenal ≥97%; (*E,E*)-2,4-octadienal ≥95%; *γ*-octalactone ≥97%; octanal ≥99%; octanoic acid ≥98%; 1-octen-3-one ≥50%; (*E*)-2-octenal ≥94%; phenylacetaldehyde ≥90%; phenylacetic acid ≥99%; 2-phenylethanol ≥99% (Sigma Aldrich, Steinheim, Germany).

References

1. Czerny, M.; Buettner, A., Odor-active compounds in cardboard. *J. Agric. Food Chem.* **2009,** *57*, 9979-9984.
